# Life history trade-offs and the partitioning of maternal investment: Implications for health of mothers and offspring

**DOI:** 10.1093/emph/eoy014

**Published:** 2018-08-16

**Authors:** Jonathan C K Wells

**Affiliations:** Childhood Nutrition Research Centre, UCL Great Ormond Street Institute of Child Health, 30 Guilford Street, London WC, UK

**Keywords:** life history strategy, pregnancy, lactation, maternal investment, non-communicable disease

## Abstract

Lay Summary: This review sets out the hypothesis that life history trade-offs in the maternal generation favour the emergence of similar trade-offs in the offspring generation, mediated by the partitioning of maternal investment between pregnancy and lactation, and that these trade-offs help explain widely reported associations between growth trajectories and NCD risk.

Growth patterns in early life predict the risk of non-communicable diseases (NCDs), but adaptive explanations remain controversial. It is widely assumed that NCDs occur either because of physiological adjustments to early constraints, or because early ecological cues fail to predict adult environmental conditions (mismatch). I present an inter-generational perspective on developmental plasticity, based on the over-arching hypothesis that a key axis of variability in maternal metabolism derives from life history trade-offs, which influence how individual mothers partition nutritional investment in their offspring between pregnancy and lactation. I review evidence for three resulting predictions: (i) Allocating relatively more energy to growth during development promotes the capacity to invest in offspring during pregnancy. Relevant mechanisms include greater fat-free mass and metabolic turnover, and a larger physical space for fetal growth. (ii) Allocating less energy to growth during development constrains fetal growth of the offspring, but mothers may compensate by a tendency to attain higher adiposity around puberty, ecological conditions permitting, which promotes nutritional investment during lactation. (iii) Since the partitioning of maternal investment between pregnancy and lactation impacts the allocation of energy to ‘maintenance’ as well as growth, it is expected to shape offspring NCD risk as well as adult size and body composition. Overall, this framework predicts that life history trade-offs in the maternal generation favour the emergence of similar trade-offs in the offspring generation, mediated by the partitioning of maternal investment between pregnancy and lactation, and that these trade-offs help explain widely reported associations between growth trajectories and NCD risk.

## INTRODUCTION

There is compelling evidence that patterns of nutrition and growth during early life shape diverse components of adult phenotype, as recognized in the developmental origins of adult health and disease (DOHaD) hypothesis [1]. While the public health implications are increasingly recognized, the evolutionary basis of developmental plasticity remains more controversial.

Many researchers consider two ‘adaptive’ models of developmental plasticity—either that it reflects developmental adjustments to resolve effects of early constraints on nutritional supply, or that it adjusts phenotype to current ecological cues in anticipation of experiencing similar conditions in adulthood [2]. However, I have agued that neither approach adequately emphasises maternal phenotype as the initial source of both resources and information received by the offspring [3, 4].

An alternative approach is to consider early developmental plasticity as a response to the magnitude and scheduling of maternal nutritional investment, thus exposing each offspring to patterns of investment that maximise maternal fitness [3–5]. Previously, I have argued that maternal phenotype (conceptualized broadly as ‘maternal capital’) represents the ‘ecological niche’ to which each new generation is exposed, and hence is the primary influence on early nutrition and developmental trajectory [3–5]. Various dimensions of maternal capital may be relevant, here I focus on one aspect by proposing the over-arching hypothesis that variability in maternal investment is shaped by life history trade-offs that emerged during maternal development. This may help explain both why certain developmental trajectories predict adult ill-health, and why profiles of both reproductive strategy and health may propagate across generations.

## NUTRITIONAL INVESTMENT IN EARLY LIFE

Nutrition is the key mechanism through which mothers invest in offspring in early life. In placental mammals, both pregnancy and lactation involve the transfer of energy, nutrients, cells, hormones and immune agents from mother to offspring. Maternal metabolism underpins both processes, but the limiting factors differ, and this has major implications for the overall profile of nutritional investment and its variability between mothers.

**Figure 1.**
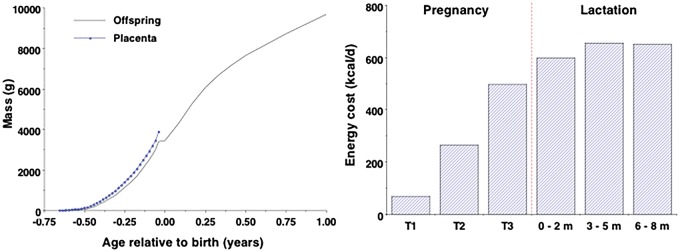
(**a**) Growth in mass of fetus/infant up to 12 months post-partum, and placenta up to term. (**b**) Energy costs of pregnancy by trimester (T) and of lactation over the first 8 months post-partum. Data from refs. [7] and [8]

In energetic terms, placental nutrition is cheaper than lactation [6, 7]. Being smaller, the fetus has substantially lower energy requirements than the infant, even after addressing placental costs [8]. Figure 1 highlights (i) the growth trajectory of the fetus/infant and placenta [8, 9], and (ii) the average maternal energy costs of pregnancy and lactation [7].

Beyond energy costs, several physiological constraints on maternal investment during pregnancy become less relevant after delivery. As discussed below, these relate to maternal metabolic turnover, body size and bio-thermodynamics during pregnancy, whereas they relate primarily to energy balance during lactation. The relationship between maternal phenotype and the magnitude of nutritional investment therefore shifts following delivery.

It might seem irrelevant how much investment is received by each offspring before versus after birth, providing that the sum total over the combined period is similar. However, the relative magnitudes of growth achieved before and after birth have major implications for adult body size, composition and metabolism, and for long-term health and demographic outcomes [10–12]. The relative ‘partitioning’ of maternal investment between fetal life and infancy is therefore a crucial issue both in evolutionary medicine and in public health.

Nutritional investment during pregnancy has unique benefits. Most rounds of cell division occur before birth [13], hence fetal life is a critical period for the structural and functional development of diverse organs and for epigenetic development [14–17]. Collectively, these traits underpin the long-term capacity for homoeostasis, and the magnitude of prenatal growth is a valuable marker of the intrinsic quality of the body, and hence likely longevity.

After birth, elevated energy supply can accelerate weight gain, however this may also generate costs, such as excess fat deposition, oxidative stress and telomere attrition [18, 19]. Rapid growth can therefore elevate ‘metabolic load’, defined as traits that challenge homeostasis [16].

Delaying growth to post-natal life therefore has very different phenotypic effects compared to growth *in utero* [10], and early growth patterns are a powerful predictor of later health status [20]. Both small size at birth and subsequent compensatory growth, especially during early childhood, are well-established risk factors for non-communicable diseases (NCDs) in adulthood [21, 22].

**Figure 2.**
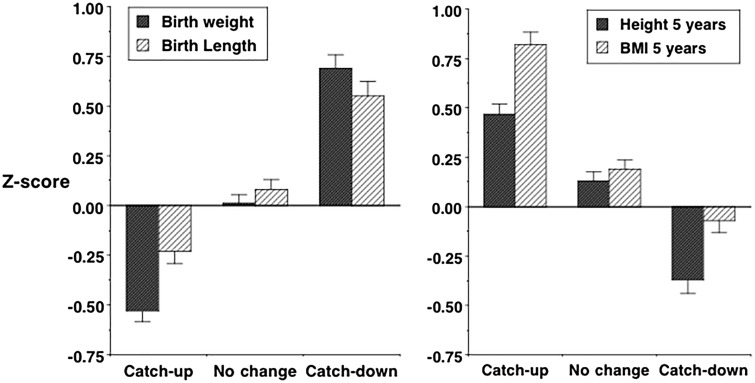
Association between infant growth pattern and (**a**) weight and length z-scores at birth and (**b**) height and BMI z-scores at 5 years. Data (mean ± standard error) from ref. [23], reproduced with permission from ref. [17]

The ‘partitioning’ of maternal investment across successive periods incorporates a dynamic element. Smaller neonates tend to undergo a degree of catch-up in early post-natal life while larger neonates show slower infant growth [23], though there is also variability. In some settings, such adjustment ‘overcompensates’: in a UK cohort, for example, infants growing rapidly tended to be smaller at birth, but taller and fatter at 5 years, while infants growing slowly showed the opposite pattern [23] (Fig. 2). Similar patterns have been observed in first-borns, typically smaller at birth compared to later-borns [24] but taller in adulthood [25]. However, in less affluent populations, post-natal catch-up is limited and may not fully compensate for small birth size [26].

## DEVELOPMENTAL VARIABILITY RECONSIDERED

From an evolutionary perspective, variability in early growth patterns can be re-interpreted within an adaptive framework. Life history theory treats energy as a limited resource, that must be allocated competitively between *maintenance*, *growth*, *reproduction* and *defence* [27]. Relative allocation patterns then represent a life history strategy, shaping a series of ‘decisions’ such as how fast to grow, when to start reproducing, how many offspring to produce, and how much to invest in each [28]. One powerful influence on each individual life history is extrinsic mortality risk: as the odds of survival decline, the optimisation of fitness favours diverting energy from growth and maintenance towards reproduction and immediate survival [29]. Exactly the same scenario applies to the intrinsic quality of the body, which likewise shapes fitness and longevity [30, 31]. Lower birth weight indicates poorer capacity for long-term maintenance [16], and hence shorter projected lifespan, and this scenario is exacerbated if compensatory catch-up occurs.

Most adaptive explanations for developmental plasticity have focused on individuals, and how they respond to diverse ecological factors [2]. However, all ecological factors during pregnancy are transduced by maternal phenotype [3, 32], indicating that maternal life history strategy is highly relevant to any adaptations made by the offspring. Paradoxically, maternal dietary intake during pregnancy has relatively modest effects on fetal growth [33–35], although supplementation promotes modest increases in birth weight among chronically under-nourished mothers [36–38]. Why, therefore, do mothers vary amongst themselves in the magnitude of investment in prenatal versus post-natal life?

This review develops the hypothesis that the *partitioning* of maternal investment between pregnancy and lactation is both a consequence of the mother’s own life history trajectory, and also a contributing factor to life history trajectory in the following generation. This generates three specific predictions:

- Mothers allocating relatively more energy to growth during their development are able to promote nutritional investment in their offspring during pregnancy.
- Mothers allocating relatively less energy to growth during their development are unable to invest as much in pregnancy, and compensate by allocating energy to fat stores, which promote nutritional investment in their offspring during lactation.
- These trade-offs affect ‘maintenance’ in both mothers and offspring, and thereby contribute to variability in NCD risk in both generations.

## MATERNAL INVESTMENT DURING FETAL LIFE

Maternal basal metabolism comprises a key constraint on the magnitude of growth attainable by the fetus [39]. Across a range of mammal species, both neonatal mass and gestation length scale allometrically with maternal weight, though the pattern varies between species with altricial versus precocial offspring [40]. Moreover, both neonatal brain mass and maternal basal metabolic rate (BMR) scale with maternal weight to the power 0.75, indicating an isometric association between maternal BMR and neonatal brain mass [39]. Overall, these studies indicate a fundamental role of maternal BMR in determining the magnitude of investment during pregnancy. Carbohydrate accounts for ∼80% of fetal fuel consumption, the remainder coming from substrates such as amino acids and free fatty acids [41].

As pregnancy progresses, however, maternal metabolism reaches an inherent limit in its ability to transfer energy to the fetus, due in part to the high glucose demands of the fetal brain [42–44]. Human birth is therefore proposed to occur at the time-point when fetal energy requirements exceed the capacity of maternal metabolism to meet that demand through placental nutrition [42, 44]. In late pregnancy, some maternal skinfold thicknesses decline, indicating that fetal growth costs exceed the energy supplied from maternal dietary intake [45, 46]. This benefits the fetal brain and enables fetal fat deposition, since fatty acids have limited capacity to cross the placenta [48].

The importance of maternal basal metabolism for fetal growth is supported by studies in diverse settings: with the exception of one small Swedish study, maternal fat-free mass (FFM), the primary site of energy and protein metabolism, is consistently reported to be a stronger predictor of offspring birth weight than maternal fat mass (Table 1). Above a certain threshold maternal fat stores may elevate birth weight, for example maternal obesity is associated with high body fat in the offspring [49], while in one African population, mothers showed a net loss of fat mass during pregnancy, indicating the diversion of energy stores to fund fetal growth [50]. In between these extremes, however, maternal adiposity appears less important for fetal investment than FFM.

**Table 1.** Associations of (a) maternal body composition with birth weight and (b) maternal adiposity with lactation

Population	*N*	BC Method	(a) Relative associations of maternal FFM and FM* with birth weight	References
*High-income*				
Ireland	2618	BIA	Birth weight increased 19.8 (95%CI 17.0–22.7) g per kg FFM, no association with FM	[115]
Ireland	254	BIA	Birth weight increased 13.7 (95%CI 0.4, 27.1) g per kg FFM, no association with FM	[116]
Ireland	184	BIA	Birth weight increased 16.3 (SE 5.0) g per kg FFM @ 28 weeks, no association with FM	[117]
Italy	29	BIA	Birth weight associated with FFM (*r* = 0.38, *P* = 0.035), not with FM (*r* = −0.02, *P* = 0.9)	[118]
USA	200	Deuterium	Birth weight increased 34.9 (SE 1.0) g per kg TBW, no association with FM	[119]
Sweden	23	Deuterium	Birth weight associated with FM (*r* = 0.49, *P* = 0.017) but not FFM (*r* = 0.26, ns)	[120]
*Low-/middle-income*				
India	76	DXA	Birth weight associated more strongly with FFM (*r* = 0.46, *P* < 0.001) than FM (*r* = 0.25, *P* < 0.05)	[121]
Chile	224	Deuterium	Birth weight associated more strongly with FFM (*r* = 0.38, *P* < 0.001) than FM (*r* = 0.27, *P* < 0.05)	[122]
Mexico	196	BIA	Birth weight increased 19.0 (SE 4.6) g per kg FFM, 9.5 (SE 5.4) g per kg FM	[123]
Sudan	1000	Anthropometry	Birth weight associated with FFM but not with SKF	[124]
Bangladesh	350	BIA	Birth weight increased 32.0 (95%CI 10.6, 53.5) g per kg TBW @ 10 weeks, no association with UAFA	[125]
China	1150	BIA	Birth weight associated with FFM in all 3 trimesters, no association with FM	[126]

Population	*N*	BC Method	(b) Changes in markers of maternal adiposity during lactation	References
*High-income*				
UK	10	Deuterium	FM declined 0.59 kg/m from birth to 4 m, then increased 0.11 kg/m from 4 to 8 m	[127]
USA	21	Skinfolds	Declines in supra-iliac and subscapular but not triceps SKF, from birth to 6 m	[128]
USA	45	Skinfolds	Declines in supra-iliac and subscapular but not triceps and biceps SKF, from birth to 4 m	[129]
Sweden	13	MRI	Thigh fat and lower trunk fat declined by 0.26 kg/m from 0.2 to 12 m, upper trunk fat by 0.07 kg/m	[130]
*Low-/middle-income*				
India	76	Anthropometry	Hip circumference fell by 1 cm/m from birth to 6 m, waist circumference declined only by 0.2 cm/m	[131]
India	35	DXA	FM in legs declined by 0.06 kg/m over 12 m from birth, negligible change in arm or trunk FM	[132]
Mexico	30	Deuterium	FM declined 0.70 kg/m from 4 to 6 m	[133]
Philippines	40	Skinfolds	Estimated FM decreased by 0.13 kg/m from 1.5 to 7 m	[134]
Guatemala	18	Skinfolds	Following lactation for ≥6 m at baseline, weight increased 0.35 kg/m over 2.5 m, highly correlated with SKF	[135]

FFM—fat-free mass; FM—fat mass; TBW—total body water, a proxy for fat-free mass; UAFA—upper arm fat area.

BIA—bio-electrical impedance analysis; DXA—dual-energy X-ray absorptiometry; MRI—Magnetic resonance imaging; SKF—skinfolds.

* Maternal body composition measured at term, unless otherwise specified in weeks of gestational age; m-month.

**Figure 3.**
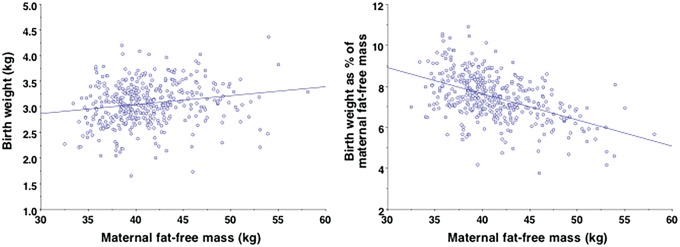
Associations between maternal fat-free mass and offspring size in an Ethiopian birth cohort (*n* = 403), for (**a**) absolute birth weight and (**b**) birth weight as a percentage of maternal fat-free mass. Andersen, Friis, Kaestel, Wells, Girma, unpublished data

Moreover, maternal body size has several other implications for the magnitude of investment during pregnancy. First, a larger FFM increases the energetic efficiency of funding fetal growth. Figure 3a plots birth weight against maternal FFM in an Ethiopian cohort, indicating that larger mothers invest more energy in absolute terms. The association is not strong, with each additional kg FFM associated with 17 g greater birth weight. In terms of efficiency, however, reproduction is relatively cheaper for larger mothers. Figure 3b plots birth weight as a percentage of maternal FFM against FFM, showing that smaller mothers invest relatively more in fetal growth. This indicates a constraint of energy transfer to the fetus in smaller mothers.

Second, ecological factors may also constrain maternal BMR. For example, BMR is around 4.5% lower in tropical populations, even after controlling for body size [51]. Lower heat production is required to maintain optimal body temperature in hotter environments, but from the opposite perspective this lower energy expenditure may constrain fetal growth [52]. Especially during physical activity, metabolism requires heat to be dissipated from the body, and this is promoted by greater surface area relative to body mass. Across populations, environmental heat stress is associated with lower adult FFM [53], and with lower birth weight after controlling for maternal height [54]. Lower maternal FFM and BMR in heat-adapted populations may therefore contribute to lower birth weights.

Third, beyond metabolic pathways, maternal height may also constrain fetal growth. The increased risk of low birth weight offspring among mothers of maternal short stature is mediated in part by reduced intra-uterine volume [55, 56]. Similarly, maternal height has been positively associated with pelvic dimensions in several populations [57–59], and there is some indication that this may impact fetal growth. This might relate in part to physiological regulatory mechanisms [60], and in part to smaller mothers deliberately ‘eating down’ in late pregnancy in order to reduce the risk of birth complications [61, 62].

In conclusion, larger mothers with greater height, FFM and skeletal dimensions are able to invest more during pregnancy than smaller mothers. The association between maternal phenotype and the capacity for nutritional investment during lactation, however, is different.

## MATERNAL INVESTMENT DURING LACTATION

Humans are ‘capital’ breeders, storing energy in advance in order to fund reproduction regardless of dietary energy availability [63]. Consistent with lactation being more costly than pregnancy (Fig. 1), the accumulation of fat tissue before/during pregnancy appears primarily to fund lactation. Breast-milk can transfer greater levels of lipid to the offspring compared to placental nutrition, thus increasing the mother’s overall capacity to meet the rising energy requirements of the offspring after birth [42, 43]. Approximately 40–50% of breast-milk energy content is provided by triglycerides, derived directly from the maternal diet, from *de novo* synthesis, or from body fat stores [64].

Prior to reproduction, women store fat disproportionately in the gluteo-femoral region, and even in populations with low average BMI, around 20–25% of body weight is fat. Additional fat accretion typically occurs during pregnancy, ranging from <1 kg in energy-constrained settings ∼3.5 kg in well-nourished populations, and up to ∼0.8 kg per month can be lost during lactation [65], again disproportionately from the gluteo-femoral region (Table 1). Among chronically under-nourished populations, both fat gains during pregnancy and losses during lactation are substantially lower [66], though successive pregnancies among chronically under-nourished women can induce cumulative weight loss, a scenario termed ‘maternal depletion’ [67].

Alongside fat oxidation, the energy costs of lactation may be met from dietary energy intake. Averaging across several studies, energy intake increased by ∼360 kcal/day by peak lactation, representing over half the costs of lactation, but again there was also substantial variability between populations [7].

Within populations, a number of studies report that mothers who are fatter or gain more energy stores during pregnancy have infants who gain more weight [68–70]. This may involve direct energy transfer during breast-feeding, for example maternal nutritional status is associated with the hormonal and nutritional content of breast-milk [71], which in turn is associated with infant growth rate and body composition [71–73]. However, hormonal programming during fetal life may also contribute, for example umbilical cord concentrations of adiponectin and leptin are likewise associated with fetal and infant weight gain and subsequent body composition [74, 75]. Again, the magnitude of maternal adiposity is related to the efficiency of lactation: to divert a given amount of energy into breast-milk is relatively cheaper for fatter compared to thinner mothers (ie, it generates a weaker trade-off against other functions), as a lower fraction of total energy stores is required [76].

Importantly, the contribution of gluteo-femoral fat to lactation relates not only to its energy content per se, but also to specific fatty acids that promote infant brain development. Beyond absolute reductions in energy stores, lactating mothers tend to redistribute fat from lower to upper body depots, allowing the diversion of fatty acids from gluteo-femoral depots to the offspring [77, 78].

Of particular relevance to the hypotheses under discussion here, substantial catch-up in weight, length and head circumference is possible in breast-fed infants born small for gestational age [79], providing that the mother has adequate energy stores or dietary supply. Chronic maternal under-nutrition may minimise such catch-up by constraining the quality and quantity of breast-milk [80, 81], though see [82].

In summary, therefore, maternal adiposity plays a key role in nutritional investment during lactation, and this contrasts with pregnancy where FFM and BMR are more important somatic factors. Why, however, do individual mothers vary in their relative investment during these two successive periods? I hypothesise that the mother’s life history is a key factor shaping the partitioning of investment, and that this generates correlated levels of energy allocation to growth and maintenance in each generation.

## MATERNAL LIFE HISTORY AND THE PARTITIONING OF INVESTMENT

According to life history theory, environments with high mortality risk and shorter life expectancy promote the allocation of energy to survival and reproduction over growth and maintenance [27, 28]. Specific predictions are that maturation is faster, final adult size is smaller, reproduction begins earlier, lifespan is shorter, ageing occurs faster, and offspring quality is traded off against offspring quantity [83]. Conversely, low-mortality environments favour investing more in maintenance (potentially extending lifespan, and hence the duration of the reproductive career) and growth (promoting the efficiency of reproduction and the quality of offspring). Specific predictions are that maturation is slower, final adult size is larger, reproduction begins later, lifespan is longer, ageing occurs at a slower rate and offspring quality is favoured over quantity. However, life history variability is not a simple ‘fast-slow’ dichotomy, rather individual mothers are distributed along continua of trade-offs, such that we can predict correlations between life history characteristics of mothers and their offspring.

The functional trade-offs that underlie life history variability are very apparent in somatic traits [17]. Increased energy allocation to growth promotes adult height and FFM, whereas allocating energy to body fat promotes survival, as adipose tissue plays multiple roles in immune function [84–87], and reproduction. Once we recognise this trade-off, we can see that it involves exactly the traits highlighted above that impact the partitioning of maternal investment between pregnancy and lactation.

For example, reducing energy allocation to growth during maternal development (resulting in short stature and reduced FFM) reduces the capacity for maternal investment during pregnancy. This equates to constraint of investment in ‘maintenance’ during early ‘critical windows’, and indicates a shorter expected lifespan and duration of the reproductive career of the offspring [17, 30]. Fetal exposure to a mother whose energy allocation strategy has favoured survival/reproduction over growth/maintenance steers the offspring to reproduce the same trade-offs.

Consistent with that, several studies have linked early growth constraint with some degree of preservation of fatness [88–91], though under the harshest conditions both height and fatness are constrained [92]. Similarly, short stature in adult women is associated with greater adiposity compared to tall women, and an enhanced tendency for weight gain in energy-rich environments (Table 2) [93], which at a mechanistic level may be mediated by reduced capacity for fat oxidation [93]. Exposure to an obesogenic environment may magnify the strength of these associations, via the tendency for obesity to provoke insulin resistance and elevate fat accretion.

**Table 2.** Associations of adult stature and infant weight gain with markers of reproductive potential

Population	*N*	Association of adult stature in women with markers of adiposity	Ref.
*High-income*			
Germany	15248	Prevalence of obesity (BMI > 30) increased inversely in association with height centile	[136]
Russia (Siberia)	59	Shorter Buryat women have higher BMI and % fat and lower fasting fat oxidation rate than taller women	[93]
Serbia	2539	Short women had higher waist circumference, BMI and waist-hip ratio than tall women, but similar hip girth	[137]
US	3815	Short stature and lower leg length were associated with higher % fat	[138]
Israel	1587	Short stature was associated with greater BMI	[139]
*Low-/middle-income*			
Brazil	1180	Short stature was associated with higher % fat and waist-hip ratio compared to women of tall stature	[140]
Brazil	48	Over 4 y, waist-height ratio increased in short mothers but not normal-height mothers (p for interaction = 0.04)	[141]
Mexico	69996	BMI was 1.2 kg/m^2^ higher in women with height <150 vs >150 cm	[142]

Population	*N*	Association of infant weight gain with age at menarche	Ref.
*High-income*			
UK	2457	Low birth weight and faster infant weight gain were associated with early menarche, with infancy the dominant effect	[143]
UK	2715	Faster weight gain from birth to 2 m and 2 to 9 m associated with higher childhood fatness and earlier menarche	[144]
Germany	87	Faster growth in infancy, but not size at birth, was associated with earlier menarche	[145]
US	262	Faster weight gain from 4 to 12 m, but not birth weight or weight gain birth to 4 m, was associated with earlier menarche	[146]
US	856	Higher birth weight and faster weight gain birth to 6 m, 6–12 m and 12–24 m were associated with earlier menarche	[147]
*Low-/middle-income*			
Philippines	997	Thinness (but not weight) at birth and faster growth from birth to 6 m was associated with earlier menarche	[148]
Brazil	2083	Lower birth weight followed by faster growth birth to 19 m and 19–43 m was associated with earlier menarche	[149]
Jamaica	140	Faster growth in infancy, but not size at birth, was associated with earlier menarche	[150]
South Africa	1201	Rapid weight gain from birth to 1 years was associated with earlier menarche and greater adult adiposity	[151]

BMI—body mass index; m—month; y—year.

In similar manner, under conditions of persistent energy scarcity both growth and maturation rate are constrained, such that if energy supply increases, menarche occurs earlier while growth also increases [94]. When post-natal energy supply is less constrained however, the developmental trade-off between growth and adiposity is mediated by maturation rate, so that early menarche is associated with higher adiposity and shorter adult height [95–97].

The inter-generational transmission of female life history strategy is evident in the UK ALSPAC cohort, where mother’s age at menarche was associated with early growth patterns, maturation rate and adolescent body composition of daughters. Mothers who had experienced earlier menarche were shorter and fatter compared to those who had experienced later menarche [98]. Their daughters showed faster rates of infant growth, earlier menarche and higher levels of body fat. Taller mothers with later menarche and lower adiposity had daughters that developed similar traits.

**Figure 4.**
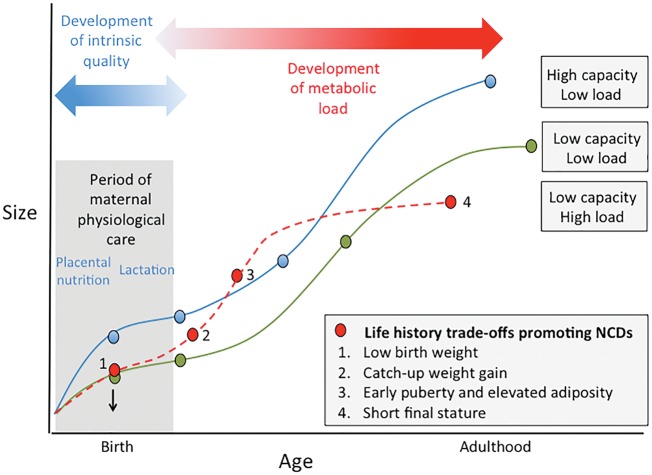
Conceptual diagram illustrating life history trajectories and their associated risk of Non-Communicable Diseases (NCDs). Each trajectory demonstrates different trade-offs, shaping both metabolic capacity and load (traits relevant to the life-course emergence of NCD risk) and the somatic traits that underlie partitioning of maternal investment between pregnancy and lactation. Blue: high maternal investment in pregnancy favours energy allocation to growth/maintenance, promoting longevity and health. Green: low maternal investment in pregnancy is followed by post-natal energy constraint, preventing catch-up. This leads to small adult size but low NCD risk. Red: low maternal investment in pregnancy is followed by catch-up during lactation, continuing into childhood. The extra energy accelerates maturation and increases adiposity without benefitting adult size, thus increasing NCD risk. Each of these trajectories produces in adult women the traits that favour the same trajectory in the next generation. Redrawn with permission from ref. [17]

To summarize this analytical framework, Fig. 4 contrasts the developmental trajectory of three hypothetical life history trajectories [17]. Greater fetal weight gain indicates the allocation of energy both to somatic growth, and to physiological traits beneficial for lifelong homeostatic maintenance and hence longevity. This increases the pay-off for extending the period of growth to attain large adult size, enabling a profile of maternal investment that favours the emergence of similar life history trade-offs in the next generation. In contrast, reduced fetal weight gain limits the allocation of energy to growth and maintenance. Under continued harsh conditions, post-natal growth remains constrained, and menarche occurs ‘early’ in terms of small body size, leading to small adult size. Should post-natal energy supply improve, it is too late to benefit maintenance. Instead, maturation accelerates so that menarche now occurs early in terms of time, accompanied by elevated fat stores, but because the growth period is shortened, adult size remains small. Through the partitioning described above, the combination of short adult stature and high fatness favour the same trade-offs emerging in the next generation.

Several studies have highlighted how greater maternal investment during infancy accelerates the pace of maturation among female offspring (Table 2). Across both high-income and low-/middle-income country populations, faster infant weight gain predicts earlier menarche, whereas birth weight, indexing maternal investment during pregnancy, either shows no such association with menarche or the reverse pattern. A study of >80 000 UK women examining the interactive associations of fetal and post-natal growth with age at menarche confirmed that post-natal growth demonstrates a stronger effect. Menarche occurred only ∼2 months earlier in those with low relative to high birth weights, but occurred a year earlier in those of high versus low weight at 7 years, regardless of birth weight [99].

In summary, the available evidence indicates that maternal somatic trade-offs between linear growth and adiposity are associated with differential partitioning of investment in the offspring between fetal life and infancy.

## INTERGENERATIONAL TRANSMISSION OF LIFE HISTORY STRATEGY AND NCD RISK

So far, this paper has argued that mothers are subject to life history trade-offs, and that this shapes developmental trajectory in the next generation. Because these trade-offs affect not only growth/reproduction but also the relative allocation to ‘maintenance’, it becomes clear that an additional connection is between life history strategy and metabolic health, best conceptualized through risk of chronic non-communicable diseases (NCDs). On this basis, since life history trade-offs are transmissible across generations, so is the risk of NCDs, strongly mediated by early growth patterns (an index of energy allocation to metabolic capacity) and subsequent adiposity (an index of energy allocation to metabolic load).

For example, a study of South Asian women in the UK showed how maternal investment in fetal life shapes the subsequent life history trajectory of daughters. Using birth weight as a simple proxy for maternal investment *in utero*, lower birth weight was associated with earlier menarche, shorter adult stature, higher levels of body fat, and higher blood pressure [30]. This indicates diverting energy to reproduction, at the expense of linear growth and homeostatic maintenance.

Another study showed how these associations become magnified when the shift between fetal and post-natal nutrition becomes more extreme. Indian girls who migrated to Sweden very early in life initially demonstrated very poor levels of growth, but nevertheless underwent precocious puberty and became short, highly adipose adults [100]. In both these studies, ample energy for catch-up growth was presumably available, thus allowing the acceleration of maturation. Similar studies have associated earlier mother’s menarche with elevated adiposity and offspring blood pressure [101–103]. Importantly, some of these inter-generational associations extend to sons as well as daughters [98, 102], though the long-term consequences for sons have received little attention.

According to the arguments above, the transmission of life history strategy across generations is expected to correlate not only with differential somatic phenotype, but also a broader range of health outcomes. Greater fetal weight gain indicates greater investment in maintenance, predicting a longer lifespan, as well as growth. We should therefore expect larger birth weight, later menarche and taller adult height all to correlate with lower NCD risk, as well as the potential to transfer the same life history strategy to the next generation. Conversely, reduced fetal weight gain followed by catch-up growth, accelerated maturation and adiposity, is predicted to superimpose a high metabolic load on a diminished metabolic capacity, resulting in elevated NCD risk (Fig. 4).

Consistent with this perspective, numerous studies have linked somatic markers of trade-offs favouring survival and reproduction over growth and maintenance with elevated adult NCD risk. Beyond well-established inverse associations with birth weight, many NCD risk factors are elevated in association with short stature, including insulin resistance, elevated blood lipids, and higher blood pressure [104–107]. Likewise, the direct association between adiposity and NCD risk in adult women is well-established [108, 109], while earlier menarche has also been associated with many NCD risk factors [110–112]. Finally, higher levels of reproductive investment appear to come at a cost to maternal maintenance, demonstrated by correlations between fertility and the risk of some NCDs. All of these associations indicate a reduced long-term capacity for homoeostasis among those favouring reproduction over growth/maintenance. However, a caveat is that hormonal profiles associated with fertility can also protect against certain cancers, hence the association between maternal fertility and morbidity/mortality varies across different diseases [113].

One final issue is to consider how ecological conditions interact with these trade-offs. Many populations have a long history of lower average birth weight, indicating limited maternal investment *in utero*, but post-natal catch-up was likely also limited [26]. Such populations are only now experiencing increased rates of NCDs in association with recent nutrition transition This is equivalent to shifting from the green to the red trajectory in Figure 4, and to shift further to the blue line, the quality of infant growth would also need to improve. This suggests that the NCD-costs of delaying maternal investment until after birth may be relatively modest, until they interact with an obesogenic setting that promotes metabolic load throughout childhood. Two large studies, from Europe and India, have linked the presence of NCDs in old age with a combination of poor early growth followed by excess BMI gains from mid-childhood onwards [21, 114]. Thus, the long-term impact of infant weight gain on NCD risk is mediated by nutritional experience after weaning, and obesogenic environments may substantially amplify the costs associated with growth variability within the critical window of lactation.

## CONCLUSION AND SUGGESTIONS FOR FUTURE STUDIES

In this review, I have developed earlier arguments to set out a new perspective on the adaptive basis of developmental plasticity. Most researchers assume either that developmental plasticity allows response to resource limitations (e.g. sacrificing one function or trait to protect another), or that it allows an adaptive response to immediate cues in anticipation of matching phenotype to adult conditions.

I have developed a different perspective, proposing that developmental plasticity incorporates adaptation of the offspring to the magnitude of maternal capital. What I add here is that the mother’s capacity to transfer capital depends on life history trade-offs that occurred during maternal development, and that the value of a given unit of capital to the offspring depends on the stage of development when it is received. Because fetal and infant growth variability have very different associations with long-term phenotype, the mediation of maternal nutritional investment by her life history trajectory generates important trade-offs in the offspring between growth, reproductive potential and health. The partitioning of maternal capital transfer between pregnancy and lactation therefore merits further attention.

This approach helps explain why life histories and health status may show some consistency across generations, without appealing to environmental ‘prediction’ or ‘anticipation’. Rather, maternal trade-offs that constrain her investment in her own growth and maintenance are proposed to steer the offspring to make similar trade-offs in the next generation. My approach differs from previous work by emphasising the mother’s response to selective pressures, rather than environmental conditions *per se*, as the primary source of developmental constraints in the offspring. This is an important difference, for two reasons. First, mothers within a population can vary substantially in their life histories, despite inhabiting a common environmental niche, due to a range of social and physical factors acting across generations. Second, mothers may vary their investment in individual offspring in order to tailor reproductive strategy to ecological conditions. For example, where extrinsic mortality risk is high, mothers may increase offspring quantity at the expense of offspring quality. A combination of reduced linear growth and diverting available energy to fat stores may contribute to such a strategy.

Relevant trade-offs between growth, maintenance, reproduction and survival traits may be tested in epidemiological studies. In an accompanying article, for example, Macintosh and colleagues apply this theoretical paradigm to bone phenotype in a sample of women in the UK. Consistent with predictions, markers of trade-offs favouring growth and maintenance (larger birth weight, later age at menarche) are associated with stronger and longer bones in adult women, indicating a lower risk of osteoporosis. In contrast, earlier menarche is associated with greater adiposity. This study therefore provides support for the hypothesis that maternal life history strategy encompasses both somatic and functional trade-offs between reproductive potential and maintenance/health. Broadly, this perspective provides an evolutionary explanation for the extensive biomedical literature linking markers of growth and maturation with NCD risk and lifespan.

More detailed measurements of maternal and offspring body composition through the periods of pregnancy and lactation would help establish with greater confidence the extent to which maternal investment is indeed partitioned between pregnancy and lactation in association with trade-offs between height and adiposity. Another key area for future work is to investigate whether such somatic and functional trade-offs apply to male as well as female life history strategies. Given the low level of nutritional investment provided by fathers, some of the associations described here for female life histories may not necessarily apply to males.

**Conflict of interest**: None declared
